# Comparative proteomic analysis of a new adaptive *Pichia Stipitis* strain to furfural, a lignocellulosic inhibitory compound

**DOI:** 10.1186/1754-6834-6-34

**Published:** 2013-03-04

**Authors:** Xue-Cai Hao, Xiu-Shan Yang, Ping Wan, Shen Tian

**Affiliations:** 1 Enzyme and Fermentation Engineering Laboratory, College of Life Science, Capital Normal University, 105 West Third Circle Road, Haidian, Beijing, 100048, China

**Keywords:** Lignocellulosic ethanol, Furfural, *P. stipitis*, Stress tolerance

## Abstract

The development of inhibitor-tolerant ethanologenic yeast is one of the most significant challenges facing bio-ethanol production. Adaptation of *Pichia stipitis* to inhibitors is one of the most efficient ways for dealing with inhibitor problems. The molecular mechanisms involved in the tolerance and adaptation of *P. stipitis* are, however, still unclear. In the present study, we developed a yeast strain from *P. stipitis* Y7 that has improved tolerance against inhibitors. We performed comparative proteomic investigations with sodium dodecyl sulfate polyacrylamide gel electrophoresis and quadrupole time-of-flight mass spectrometry. These investigations gave insights into the tolerance of yeast strains to biomass conversion inhibitors at the protein level. Many proteins involved in glycolysis, the pentose phosphate pathway, and the tricarboxylic acid (TCA) cycle were found to be differentially expressed due to the presence of furfural. Quantitative real-time reverse transcription-PCR (RT-PCR) and metabolite analysis were utilized to provide orthogonal evidence for the results obtained. Our results provide a deeper understanding of the molecular mechanisms involved in the response of *P. stipitis* to furfural. These findings will benefit the design and development of inhibitor-tolerant yeast.

## Introduction

The continued use of fossil fuels as a principal source of energy has led to a variety of environmental, economical and political concerns: as such, research into improving alternative and renewable energy strategies is of great importance. Bioethanol is one potentially viable alternative energy source. Bioethanol that is produced through the fermentation of lignocellulosic biomass from agricultural by-products, forest residues or energy crops offers many potential advantages in comparison to sugar- or starch-derived bioethanol, from both energetic and environmental points of view. Current methods of producing such bioethanol are not efficient, however, and the technology required in order to be able to use lignocellulosic biomass as a fermentation substrate faces several main challenges. One such challenge is the need for a robust fermentative microorganism that can tolerate the inhibitors present during lignocellulosic fermentation.

Inhibitors formed by acid-catalyzed hydrolysis of lignocelluloses, which include furan derivatives, weak acids, and phenolic compounds, reduce both the growth rate and fermentation of ethanologenic microorganisms [1]. Several different detoxification methods have been described and the effects on the chemical composition of lignocellulose hydrolysates have been investigated. Although detoxification improves the production of fuel ethanol, the fermentative activities, it is desirable, for economic reasons, to limit the number of additional steps required for detoxification. The development of inhibitor-tolerant ethanologenic yeast is therefore highly desirable for bioethanol production, but remains a major challenge.

*Pichia stipitis* is a well-studied, native xylose-fermenting yeast. The adaptation of *P. stipitis* to inhibitory hydrolysates is one of several possible strategies for dealing with inhibitor problems, offering an alternative to the use of detoxification methods. We previously developed a yeast strain from *P. stipitis* Y7 with improved tolerance against inhibitors that was able to ferment non-detoxified steam-exploded corn stalk with sufficient ethanol yield [2]. Further understanding of cellular stress responses to the individual inhibitor will enable the development of more tolerant strains as well as rapid and efficient fermentation of the hydrolysates.

Furfural is one of the major inhibitors in lignocellulosic hydrolysates. Many studies have shown that furfural can be converted by yeast to furfural alcohol [3-5]. The genetic mechanisms involved in furfural tolerance have been thoroughly researched. Through gene cloning and enzyme activity study, Liu et al. [6] found that the conversion of furfural is catalyzed by multiple aldehyde reductases. Traditional methods, such as metabolite, enzyme activity analysis, and kinetic analysis, can, however, analyze only one or a few metabolites, proteins or genes and are unable to globally assess the inhibition issue, which is complex and systematic [7]. The integration of different “omics” tools into the study of systems biology, including transcriptomics, proteomics, and metabolomics, provides an increasingly rich understanding of the response of microorganisms to various environmental perturbations [8,9].

In the present study, comparative proteomic investigations with sodium dodecyl sulfate polyacrylamide gel electrophoresis and quadrupole time-of-flight mass spectrometry (Q-TOF MS) were performed, in order to give insights into the tolerance of ethanologenic yeast strains to biomass conversion inhibitors at the protein level,. These studies were performed with the aim of systematically identifying proteins through the use of adapted *P. stipitis* strain Y7 and to quantify the cells treated with furfural compared with control cells under oxygen-limited conditions. Quantitative real-time reverse transcription-PCR (RT-PCR) and metabolite analysis were utilized to provide orthogonal evidence for the comparative proteome results.

## Results and discussion

### Adaptation

The evolution of *P. stipitis* Y7 was encouraged using a directed adaption strategy incorporating specifically designed adaption media. The parent *P. stipitis* strain was challenged in the adaptation medium by increasing the concentration of furfural. After adaption of *P. stipitis* to the adaptation medium with 40 mM furfural for fifty subcultures, a colony was isolated on solid medium and thereafter this strain was designated the adapted culture Y7-1. It was not possible to identify, macroscopically, any obvious gross phenotypic differences between the adapted culture Y7-1 and the parental culture Y7.

**Figure 1:**
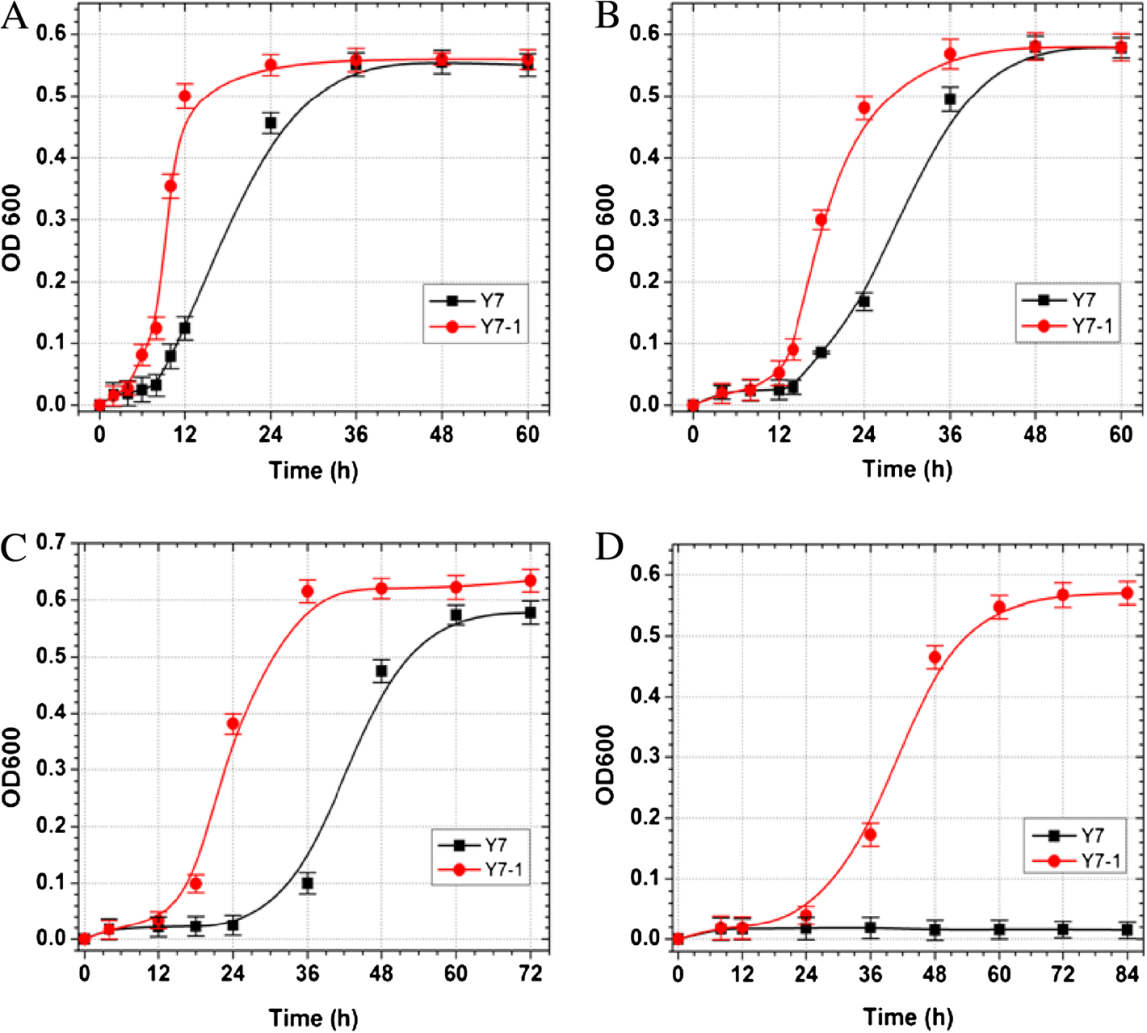
**Cell growth of parental strain Y7 and adapted strain Y7-1 as measured at OD**_**600 **_**on the defined medium containing 10 Mm (A), 20 mM (B), 30 mM (C) and 40 mM (D) furfural.**

Figure 1 shows the growth of strains Y7-1 and Y7 in the defined medium, exposed to 10, 20, 30, and 40 mM furfural respectively (A, B, C, and D). At 10 mM, Y7 showed a lag phase of 8 h. However, Y7-1 had only a 4 h lag time of cell growth and grew quickly into stationary phase in 24 h. For cultures growing in 20 mM furfural-treated media, the lag time extended to 14 h and 8 h for strains Y7 and Y7-1, respectively. In the presence of 30 mM furfural, the adapted strain had a 12-h lag phase and the parent strain had lag phases of 24 h. Under exposure to 40 mM furfural, the adapted strain grew into the logarithmic phase in 36 h. At the same concentration, the parental strain showed no substantial cell growth.

### Batch fermentation on synthetic medium and non-detoxified enzymatic hydrolysate

Table 1 summarizes the fermentation results for the defined medium containing either 30 mM furfural or the enzymatic hydrolysate of non-detoxified steam-pretreated corn stover for both the parent strain and the adapted *P. stipiti*s.

**Table 1:** **Summaries of fermentation results from the parental and the adapted strains of *P. stipitis***^**a**^

	** Defined medium containing 30 mM furfural**		**Enzymatic hydrolysate of non-detoxified steam-pretreated corn stover**	
	**Parent**	**Adapted**	**Parent**	**Adapted**
Ethanol yield on substrate (g_p_ g_s_^-1^)	0.36 ± 0.02	0.40 ± 0.01	0.36 ± 0.01	0.43 ± 0.03
Theoretical yield (%)^b^	71.06 ± 0.02	78.14 ± 0.07	69.74 ± 0.01	83.33 ± 0.03
Ethanol productivity (g_p_L^-1^ h^-1^)	0.30 ± 0.03	0.41 ± 0.04	0.28 ± 0.05	0.41 ± 0.03
Fermentation time (h)	60	48	60	48
Maximum ethanol concentration(g_p_L^-1^)	17.76 ± 0.03	19.53 ± 0.04	16.80 ± 0.05	19.87 ± 0.03
Total sugar consumption rate (g_l_^-1^ L^-1^ h^-1^)	0.82 ± 0.04	1.02 ± 0.05	0.79 ± 0.04	0.98 ± 0.06

^a^ The initial cell density for fermentation was 2.0gL^-1^.

^b^ Theoretical yield ethanol from glucose is 0.51g_p_g_s_^-1^; theoretical yield (%) calculated as ethanol yield × 100 divided by 0.51.

The adapted *P. stipitis* Y7-1 showed a good fermentation performance regardless of whether ethanol production was carried out on the medium or on the hydrolysate. The ethanol yield and productivity of Y7-1 fermented on the enzymatic hydrolysate of non-detoxified stem-pretreated corn stover were 0.43 ± 0.03 g_p_ g_s_^-1^ and 0.41 ± 0.03 g_p_ l^-1^ h^-1^, which represent increases of 19% and 46%, respectively, compared to the parent strain. Similarly, the results from the defined medium fermentation by *P. stipitis* indicated that ethanol productivity was increased from 0.30 ± 0.03 g_p_ l^-1^ h^-1^ to 0.41 ± 0.04 g_p_ l^-1^ h^-1^ after strain adaptation. This suggested that inhibitor tolerance was substantially enhanced in the adapted *P. stipitis* strain.

### Proteins differentially expressed in response to furfural

The adapted yeast strain Y7-1 exhibited significant differences in growth and glucose metabolism compared to the parent yeast in the presence of furfural during the ethanol fermentation process. Many studies have reported that abundant changes in the expression levels of proteins, caused by the presence of furfural, were localized to most compartments and were found to be involved in almost all of the functions and pathways in yeast cells, suggesting that the response of yeast to furfural is global and systematic [7,10].

In this study, the proteomic response of a *P. stipitis* strain following treatment with furfural was explored using LC-Q-TOF/MS. Proteins were identified through database searching and a total of 398 proteins were quantified. The relative protein expression levels were determined by using label-free MS quantification methods. As shown in Table 2, most of the differentially expressed proteins were involved in carbohydrate metabolism, most notably the glycolysis pathway and the alcohol catabolic process.

**Table 2:** Biological process analyses of the differentially expressed proteins according to Gene Ontology

** GO.ID**	**Term**	**Significant**	**p_value**
GO:0044262	cellular carbohydrate metabolic process	23	0.00013
GO:0005996	monosaccharide metabolic process	19	0.0003
GO:0006066	alcohol metabolic process	19	0.0003
GO:0006091	generation of precursor metabolites and energy	19	0.0003
GO:0019318	hexose metabolic process	19	0.0003
GO:0006006	glucose metabolic process	18	0.00054
GO:0044282	small molecule catabolic process	15	0.00064
GO:0006007	glucose catabolic process	14	0.00116
GO:0016052	carbohydrate catabolic process	14	0.00116
GO:0019320	hexose catabolic process	14	0.00116
GO:0044275	cellular carbohydrate catabolic process	14	0.00116
GO:0046164	alcohol catabolic process	14	0.00116
GO:0046365	monosaccharide catabolic process	14	0.00116
GO:0009056	catabolic process	21	0.00127
GO:0005975	carbohydrate metabolic process	25	0.00646

After 4 h of furfural-treated cultivation, yeast cells were harvested during the lag phase, with the analysis showing that 17 proteins (Glk1p, Tdh1p, Pgk1p, Eno1p, Gnd1p, Adh1p, Adh3p, Adh4p, Adh6p, Cit1p, Tal1p, Idh1p, Idh2p, Fum2p, Aco1p, Gre2p, Mdh1p) had been dramatically up-regulated in Y7-1, all of which are involved in glycolysis (EMP), the pentose phosphate pathway (PPP), and the tricarboxylic acid (TCA) cycle.

The expression of Glk1p, Pgk1p and Eno1p was significantly up-regulated. The expression levels of PFK2p, PDC1p, and PYK1p were not noticeably affected. It is assumed that glycolysis of the strain Y7-1 was activated following the up-regulated expression of Glk1p, Pgk1p and Eno1p, which played an increasingly important role in the glycolysis pathway. The suggestion that glycolysis is activated in the presence of furfural is supported by the results of a previous study of the conversion of furfural in aerobic and anaerobic bath cultures of *S. cerevisiae* CBS 8066 growing on glucose. This study showed that glycolysis-inducing changes in abundance are immediately affected by furfural and that the reduction of furfural relied on active glycolysis [8]. The significantly induced expression of Tdh1p, Adh1p, Adh3p, Adh5p and Adh6p demonstrates that the reduction of furfural to furfural alcohol is likely catalyzed by alcohol dehydrogenases (ADHs) and multiple aldehyde reductases [11-13].

The pentose phosphate pathway (PPP) is an important carbohydrate metabolism pathway and is the main source of cytoplasmic NADPH. The expression levels of Gnd1p and Tal1p were found to be noticeably up-regulated, indicating that *P. stipitis* tolerance to furfural may be associated with activation of the initial stage of PPP. This suggestion is consistent with reports that gene deletion mutants of the pentose phosphate pathway (PPP) genes *ZWF1*, *GND1*, *RPE1* and *TKL1* exhibited growth deficiency in the presence of furfural [14].

The TCA cycle is a central metabolic pathway, generating ATP and NADH under aerobic conditions and also producing precursors for many compounds including some amino acids. The up-regulation of Cit1p, Idh1p, Idh2p, Fum2p, Aco1p and Mdh1p revealed that the TCA cycle can be activated in the presence of furfural to produce more NAD(P)H, which allows the reduction of furfural. It has been reported that the presence of furfural in the medium results in a 50% increase in the specific rate of the TCA cycle compared to the rate seen in furfural-free medium [15].

Although it has been mentioned before that the altered expression levels of most proteins catalyzing the reactions of the central carbon metabolism (with the exception of those involved in PPP) due to the addition of furfural can lead to the rearrangement of the central carbon metabolism in *S. cerevisiae* cells, which induces tolerance of furfural, there has been no previous study focusing on the stress effects caused in *P. stipitis* by the presence of furfural [7]. Our data suggest that *S. cerevisiae* and *P. stipitis* possess similarities in the process of converting furfural by activating the central carbon metabolism (EMP, PPP and TCA).

There are some proteins related to stress responses were also changed in the furfural-treated cultures. These proteins are involved in the response of yeast to oxidative stress, superoxide metabolic process, cell redox homeostasis. The superoxide dismutase Sod1p, glutathione peroxidase Gpx2p, thioredoxins Trx1p, and quinine oxidoreductase Qor1p were significantly up-regulated. However, expression of peroxisomal catalase Cat1p was down-regulated. The oxidative stress may be related to the decrease of the NAD^+^, and NADH levels. Although we do not clearly understand the mechanisms behind the response of these proteins to furfural, the different expression patterns of the proteins related to the stress response show that furfural may present a complex stress environment to the yeast cells which affects the expression levels of proteins related to stress response [10].

### The quantitative RT-PCR results for the selected genes

**Figure 2:**
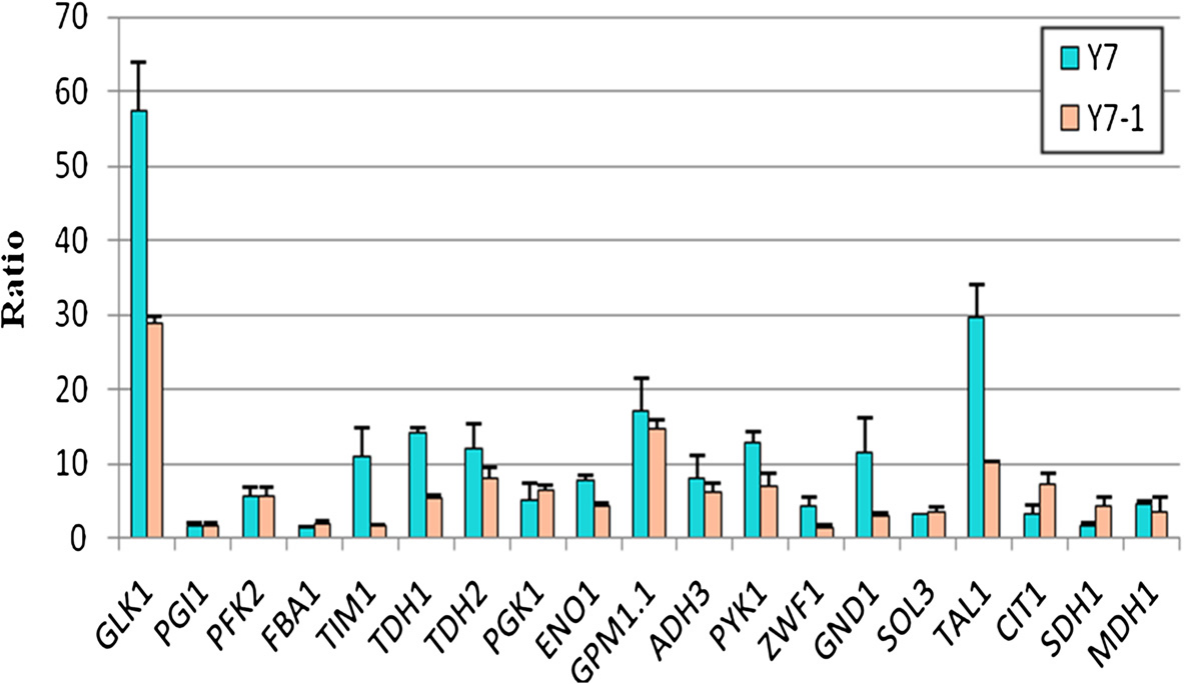
**Comparison of transcript levels for selected genes in Y7 and Y7-1.** The two strains were cultivated in flasks containing 100 ml of the YPD medium (containing 10 mM of furfural·liter^-1^) at 30°C and 150 rpm for 4 h. Mean values are presented with error bars representing variations of two standard deviations. The fold change was determined by the 2^-ΔΔCt^ method.

Although the protein expression level is influenced by protein turnover and post-translation modifications, the quantitative RT-PCR results provide orthogonal evidence of the reliability of the relative quantitative protein expression results. The transcript levels of the selected genes were measured by quantitative RT-PCR at 4 h, 6 h and 10 h. As shown in Figure 2, at 4 h after furfural treatment, Y7-1 displayed gene expression profiles distinct from the control Y7. At least 12 genes, including *GLK1*, *TIM1*, *TDH1*, *TDH2*, *ENO1*, *GPM1.1*, *ADH3*, *PYK1*, *ZWF1*, *GND1*, *TAL1*, and *MDH1* demonstrated significantly higher levels of mRNA transcripts compared to that of the parental strain Y7. Many of these genes, such as *TIM1*, *ZWF1*, *GND1*, and *TAL1,* showed 3-fold enhanced expression. *GND1* and *TIM1* showed more than 5-fold enhanced expression. The quantitative RT-PCR results for *GLK1*, *GND1*, *TAL1*, *PGI1*, *PFK2*, *ENO1* and *MDH1* were consistent with the relative quantitative protein expression results. There were also discrepancies between the quantitative RT-PCR results and the relative quantitative protein expression results for genes such as *PGK1*, *PYK1*, *ADH1*, *TDH3* and *CIT1*. It was also found, in both strains that many genes involved in the glycolysis and pentose phosphate pathways were repressed.

**Figure 3:**
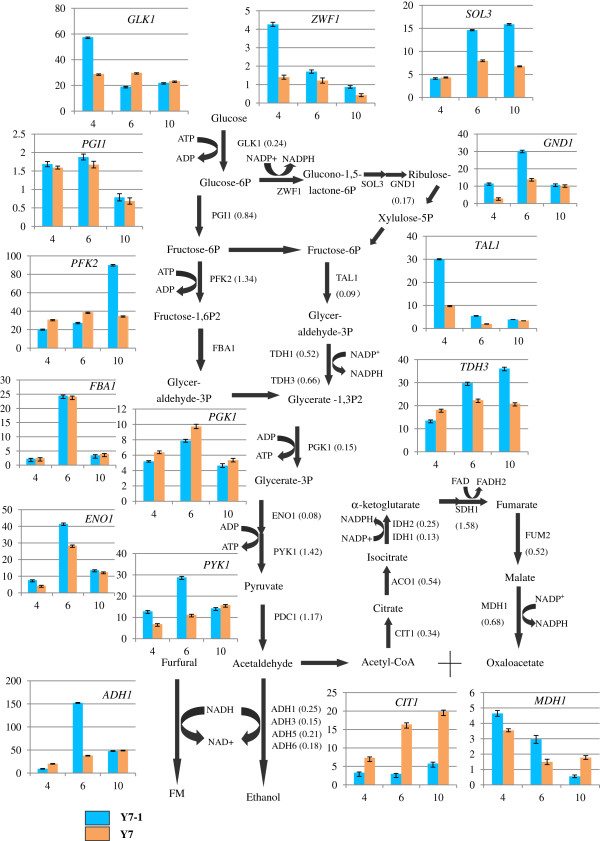
**Relative expression levels of proteins involved in the central carbon metabolism, including glycolysis, the TCA cycle and the PPP.** The ratios of proteins are presented for Y7 and Y7-1 at the 4 h time point (the value in parentheses). Comparative mRNA expressions of genes at 4 h, 6 h and 10 h time point are presented. Ratios between Y7 and Y7-1 levels are shown (column configuration).

The response of yeast to furfural is a dynamic and complex process. As shown in Figure 3, at 4 h after furfural treatment, there was no significant difference in the expression of *PGI1*, *PFK2* and *FBA1* in either strain. The expression of *GLK1*, *ZWF1*, *GND1* and *SOL3* was up-regulated for Y7-1. The pentose phosphate pathway became the activated path for glucose metabolisms and furfural conversions for Y7-1 at the 4 h time point. Glycolysis and the pentose phosphate pathway are major routes for glucose metabolisms that provide energy and important intermediate metabolites for biosynthesis and ethanol production. Unfortunately, important enzymes involved in glycolysis were inhibited by furfural in the initial stage. It has been reported that NAD(P)H dependent aldehyde reductions, which involve multiple genes, is one mechanism through which the detoxification of furfural and HMF occurs [6]. The enhancements in these expressed genes are therefore significant for NAD(P)H regenerations in order to supply the cofactors that are needed for acetaldehyde conversion and for the reduction of furfural to occur. *GND1*, *TAL1*, *ENO1*, *PYK1*, *ADH1* and *MDH1* displayed high activation levels for Y7-1 at the 6 h time point in the presence of furfural.

The expression of many genes changed at the 4 h and 6 h time points. Y7 showed continued inhibition in cell growth and in the expression of many genes at the 6 h time point. Y7-1 was able to recover from the inhibitor challenge quickly within 4 h. Transcription levels were low at 4 h for numerous genes (*PGI1*, *FBA1*, *ENO1* and *ADH1*) in glycolysis and the pentose phosphate pathway for both strains. In Y7-1, many of these genes recovered to normal or near normal function levels at 6 h. In contrast, in Y7, many genes continued to be repressed by the furfural at 6 h. At 10 h after the furfural treatment, both strains recovered their normal growth patterns. Transcription levels of most genes in Y7-1 and Y7 displayed similar conditions at 10 h, except *PFK2*, *SOL3*, and *TDH3*, which remained up-regulated in Y7-1.

Furfural conversion to furan methanol and HMF to furan dimethanol are NAD(P)H-dependent reduction activities that are controlled by multiple enzymes [16-18]. In our study, we investigated transcription dynamics during the lag phase in a major pathway involving glucose metabolism under furfural stress. Our results indicate that Y7-1 was able to withstand and quickly detoxify the furfural stress *in situ*, whilst producing ethanol, through both transcription responses and altered metabolic pathways.

## Conclusion

The addition of furfural to the strain medium inhibits biomass growth, glucose consumption and ethanol production. Based on observations of a dose-dependent yeast response to furfural, we developed a directed adaptation method and generated an adapted strain Y7-1. We evaluated the adapted ethanologenic yeast strain and demonstrated, under controlled conditions, significantly higher levels of tolerance to furfural compared with the parental strain. Adaptation can, therefore, be an effective means through which to improve microbial strains in this regard. The adapted, more tolerant, Y7-1 strain shows relatively high levels of tolerance to single inhibitors. The Y7-1 strain has also been tested against inhibitor complexes in biomass hydrolysate and has shown promise for use in biomass fermentation applications.

The proteomic data presented here provides a deeper understanding of the molecular mechanisms involved in the adaptation and response of *P. stipitis* to furfural. It was found, for example, that many proteins involved in glycolysis, the pentose phosphate pathway and the tricarboxylic acid (TCA) cycle were differentially expressed in the presence of furfural. It was found that furfural not only influences *P. stipitis* with respect to primary carbonate metabolic pathways but also causes the formation of a complex stress environment in yeast cells. Quantitative RT-PCR was utilized to provide orthogonal evidence supporting the comparative proteomics results. Our results suggest that continued efforts towards developing stress tolerant ethanologenic yeast to support a sustainable lignocellulosic biomass-to-ethanol industry are warranted.

## Materials and methods

### Strains, media, and cultivation conditions

*Pichia stipitis* Y7 is a strain that has recently been developed in our laboratory (Patent No.200810223301.5, CGMCC 2661). The detailed ethanol production profiles for *Pichia stipitis* Y7 have been reported elsewhere [2]. The yeast was cultured on a YPD plate containing 10 g/L yeast extract, 20 g/L peptone, 50 g/L glucose, and 20 g/L agar. The Y7 colony was grown in filter-sterilized media containing 10 g/L yeast extract, 20 g/L peptone, and 20 g/L glucose. The inoculate culture was prepared using freshly grown cells harvested at the logarithmic growth phase and incubated with agitation of 150 rpm for 18 h at 30°C. Cell growth was determined by standard curves that related 600 nm absorbance to cell concentration (Agilent 8453, UV-visible Spectroscopy system, Agilent Technologies, Santa Clara, CA, USA).

### Adaptation experiment

A directed adaptation method was developed and applied. *P. stipitis* Y7 strain was first maintained and cultured by sequentially transferring and growing cells in a medium containing low concentrations(10 mM/L) of furfural supplemented with 10 g/L yeast extract, 2 g/L peptone, 20 g/L glucose. No additional nutrient salts or vitamins were added to the adaptation media. The inoculated culture was grown at 30°C with agitation at 150 rpm on an orbital shaker. As the logarithmic growth phase was reached, surviving microorganisms were transferred to a fresh adaptation medium with a higher concentration (the concentration was gradually increased to 10 mM, 20 mM, 30 mM and 40 mM furfural liter-1) This iterative process was repeated for higher inhibitor concentrations until the adapted culture was continuously subcultured across more than 50 subcultures. Following this process, the adapted strain Y7-1 was obtained.

### Batch fermentation

The fermentation profiles of adapted *P. stipitis* were determined and compared with the parent strain by using inhibitor-treated synthetic medium and non-detoxified enzymatic hydrolysate. The defined medium contained 50 g/L of glucose, 10 g/L of yeast extract, 2 g/L of peptone, and was supplemented with 30 mM furfural. Enzymatic hydrolysate was produced from a pretreated solid cellulosic substrate of stem-exploded corn stover. (The steam-exploded pretreated corn stover was provided by the Henan Tianguan Group Co., Ltd (Henan, China) and contained 42.3% glucan, 0.16% furfural, 5.1% acetic acid). The pretreated solid substrate was diluted with sodium acetate buffer solution (pH 4.8) to 15% (w/v) concentration. A mixture of Celluclast 1.5 L with an activity loading of approximately 15 FPU/g cellulose and Novozyme188 with an activity loading of approximately 20 IU/g cellulose was used. Additional nutrients were then added (yeast extract of 1 g/L and peptone of 2 g/L). After 48 hours of hydrolysis at 50°C in a shaker set at 150 rpm, fermentation occurred. All fermentation experiments were performed at 30°C, 90 rpm. The working volume was 100 mL and the initial cell concentration was 2 g/L. Samples were centrifuged at 10,000 rpm for 5 min and were stored at −4C until analyzed for sugar and ethanol.

Sugar concentration was measured using a high-performance liquid chromatography (HPLC) equipped with a KNAUER NH_2_ column (5-mm particle size, 250 mm × 4.6 mm) and a KNAUER RI detector (model K-2301). Samples were run at a temperature of 30°C with a mobile phase of acetonitrile/ultrapure water at a flow rate of 1 ml/min. Ethanol analysis of the fermentation broth was carried out using a gas chromatograph (GC, model 7890A, Agilent Technologies) through a headspace sampler (HS, model 7694E, Agilent Technologies) using an external standard for calibration. The chromatograph was equipped with a flame ionization detector and an Agilent HJ-PEG column of 30 m with an internal diameter of 0.32 mm. Samples were run under the following conditions: column oven at 120°C, front injection port at 200 C, with N_2_ as the carrier gas at a flow rate of 4 ml/min.

### Sample preparation for proteomic analysis

For the proteomic analysis, the parent strain Y7 and adapted strain Y7-1 were cultivated in flasks containing 100 ml of the YPD medium (containing 10 mM of furfural) at 30°C and 150 rpm for 4 h. Cells were harvested by centrifugation. To cause cell disruption, the cells were washed twice with cold water and suspended in 1 ml of lysis buffer consisting of 20 mM Tris–HCl (pH 7.5), 250 mM sucrose, 1% Triton X-100, and 10 mM EDTA. Next, 1 mM DTT and 1 mM PMSF were added and sonicated (2 s/2 s, 5 min). The cell suspension was then vigorously mixed twice for 10 min and centrifuged in order to remove the undisrupted cells and debris and to obtain the crude extract. Supernatants were mixed with four volumes of 50% TCA-acetone at −20°C for at least 2 h or preferably overnight. The precipitate was washed twice with ice-cooled acetone, and centrifuged at 17,000 × g for 15 min. The dried cytosolic proteins were dissolved in 40 μl rehydration buffer (7.0 M urea, 2.0 M thiourea, 4.0% (m/v) CHAPS, 50 mM dithiothreitol (DTT), 0.5% (v/v) immobilized pH gradient (IPG) buffer pH 4–7 (GE Healthcare) and small amounts of bromphenol blue). Protein content was determined according to the Bradford method.

### SDS polyacrylamide gel electrophoresis and in gel digestion

The yeast protein samples were boiled for 5 min and then centrifuged. The yeast protein samples (20 μg of each sample) were mixed with loading buffer and separated on 12.5% SDS polyacrylamide gel electrophoresis gels. Electrophoresis was then performed until the dye front reached the bottom of the gel. Gels were stained using Coomassie brilliant blue R250. Each experiment was repeated at least twice.

Each of the protein bands from the stained polyacrylamide gels were excised and transferred into a 1.5 ml centrifuge tube. The excised spots were destained by incubation in 100 μl of 25 mM ammonium bicarbonate/50% acetonitrile (more solution was added as necessary in order to immerse the gel particles) and were then vortexed for 10 min. This wash/dehydration step was repeated up to 3 times. Dehydrated gel pieces were produced by adding acetonitrile (100 μl), and then submitting them to air-drying for 5–10 min. Sufficient 10 mM DTT solution was then added to cover the gel pieces and was reduced for 1 h at 56°C. The same volume of 55 mM iodoacetumide solution was added to replace the DTT solution, and the sample was incubated for 45 min at room temperature in the dark with occasional vortexing. The gel plugs were then rehydrated in 10 μl of trypsin solution (0.1 mg/ml). Digestion was performed at 37°C for 12 to 16 h. Sonication was used to extract digested peptides in the gel. Two additional extractions using 200 μl of 5% FA/50% acetonitrile were performed. Each extracted sample was mixed in a tube and dried using vacuum freeze-drying.

### Metabolome analysis by LC-Q-TOF/MS

Liquid chromatography coupled to time-of-flight mass spectrometry (LC-Q-TOF/MS) analysis was then performed on sample sets of parental and adaptive yeast strains. Samples were analyzed in a 4800 Proteomics Analyzer MALDI-TOF/TOF mass spectrometer (Applied Biosystems, Framingham, MA), in the m/z range 850–4000, in reflectron mode, with an accelerating voltage of 20 kV and with a delayed extraction set to 120 ns. All MS spectra were internally calibrated with peptides from trypsin autolysis. The MS analysis by MALDI-TOF/TOF mass spectrometry produces peptide mass fingerprints and the peptides observed with a Signal to Noise greater than 20 were collated and represented as a list of monoisotopic molecular weights.

Proteins ambiguously identified by peptide mass fingerprints were subjected to MS/MS sequencing analysis. Suitable precursors were selected from the MS spectra for the MS/MS analysis with CID on (atmospheric gas was used), using the 1 Kv ion reflector mode and a precursor mass Windows ± 5 Da. The plate model and default calibration were optimized for the MS–MS spectra processing. For protein identification, the UniProt Knowledgebase Release 14.6 (UniProtKB/Swiss-Prot Release 56.6 of 16-Dec-2008, Uni-ProtKB/TrEMBL Release 39.6 of 16-Dec-2008) was searched using the MASCOT search engine v.2.1 (Matrix Science, London; http://www.matrixscience.com) through the Global Protein Server Explorer software v3.6 from Applied Biosystems.

### Real time PCR

Y7 and Y7-1 were incubated on YPD medium (containing 10 mM of furfural · liter-1). Yeast cells were harvested periodically starting from 4 h and then at 6 h and 10 h. RNA was isolated by the Trizol method (Invitrogen, Carlsbad, CA). RNA cleanup was performed using a Qiagen RNeasy mini kit (Qiagen Sciences, MD). 10 μg of total RNA was treated with DNase (Turbo-Free, Ambion) following the manufacturer’s protocol, with the exception that an additional 1 μl of enzyme was added after 20 min of incubation at 37°C for an additional 20 min. The concentration of total RNA was determined by measuring absorbance at 260 nm (A260), with the quality of the RNA being assessed by determining the ratio of A260 to A280 and the use of agarose gel electrophoresis. Samples with RNA purity greater than 1.8 were used. cDNA was synthesized from 0.5 μg total RNA using the Prime Script TM RT reagent kit (Perfect Real Time, TaKaRa) following the manufacturer’s protocol.

The primers used in this study were designed using the free Primer 3 software. SYBR Premix Ex Taq TM (Perfect Real Time, TaKaRa) was applied for each qRT-PCR reaction. For each reaction, a total of 20 μl was used, consisting of 10 μl 2X SYBR Green Master Mix, 0.6 μl each of forward and reverse primer (10 μM), 1 μl cDNA template and 7.8 μl H2O. Amplifications were performed under the following conditions: 95°C for 3 min; 40 cycles of 95°C for 5 s, 58°C for 15 s, and 72°C for 15 s. At the end of the amplification cycle, a melting analysis was conducted to verify the specificity of the reaction. This was carried out by heating the amplification products from 55°C to 95°C at 0.5°C/10 s and monitoring the decrease in fluorescence.

Expression levels were quantified by using a TaKaRa SYBR Green Real-Time PCR Master Mix (TaKaRa) in the iQ5 Real-Time PCR Detection System (Bio-Rad, USA), which was normalized using *TUB2* expression levels as a reference. The fold change was determined by the 2^-ΔΔCt^ method.

## Abbreviations

TCA: Tricarboxylic acid cycle; Q-TOF MS: Quadrupole time-of-flight mass spectrometry; ADHs: Alcohol dehydrogenases; PPP: Pentose phosphate pathway; EMP: Embden-Meyerhof-Parnas pathway; HMF: Hydroxymethyl-furaldehyde; NADPH: Nicotinamide adenine denucleotide phosphate reduced form; NADH: Nicotinamide adenine denucleotide reduced form

## Competing interests

The authors declare that they have no competing financial interests.

## Authors’ contributions

ST and XSY conceived the project, designed the experiments, and wrote the manuscript. XCH and PW conducted the experiments and analyzed the data. ST supervised the project. All authors read and approved the final manuscript.
